# Glucose transporter type 1 deficiency syndrome and the ketogenic diet

**DOI:** 10.1002/jimd.12175

**Published:** 2019-11-13

**Authors:** Marit Schwantje, Lilly M. Verhagen, Peter M. van Hasselt, Sabine A. Fuchs

**Affiliations:** ^1^ Department of Metabolic Diseases, Wilhelmina Children's Hospital University Medical Center Utrecht Utrecht The Netherlands; ^2^ Department of Infectious diseases and Immunology, Wilhelmina Children's Hospital University Medical Center Utrecht Utrecht The Netherlands

**Keywords:** dietary treatment, GLUT1DS, ketogenic diet, SLC2A1 mutation, treatment effects

## Abstract

Glucose transporter type 1 deficiency syndrome (GLUT1DS) is characterised by deficient glucose transport over the blood‐brain barrier and reduced glucose availability in the brain. This causes epilepsy, movement disorders, and cognitive impairment. Treatment with ketogenic diet provides ketones as alternative energy source. However, not all GLUT1DS patients are on dietary treatment (worldwide registry: 77/181 [43%] of patients). The current 25‐year experience allows evaluation of effects and tolerability of dietary treatment for GLUT1DS. To this end, literature was searched up to January 2019 for individual case reports and series reporting (side) effects of dietary treatment for GLUT1DS. Upon aggregation of data for analysis, we identified 270 GLUT1DS patients with dietary treatment with a mean follow‐up of 53 months. Epilepsy improved for 83% of 230 patients and remained unchanged for 17%, movement disorders improved for 82% of 127 patients and remained unchanged for 17%, and cognition improved for 59% of 58 patients and remained stable for 40%. Effects on epilepsy were seen within days/weeks and were most pronounced in patients with early treatment initiation. Effects on movement disorders were noticed within months and were strongest in patients with higher cerebrospinal fluid‐to‐blood glucose ratio. Although side effects were minimal, 18% of 270 patients reported poor compliance. In individual patients, symptoms deteriorated upon low ketosis, poor compliance, or treatment discontinuation. Based on the good tolerability and strong favourable effect of dietary treatment on GLUT1DS symptoms, we advocate dietary treatment in all GLUT1DS patients and prompt diagnosis or screening to allow early treatment.

**Abbreviations**
CSF: cerebrospinal fluid
GLUT1DS: glucose transporter type 1 deficiency syndrome
IQ: intelligence quotient
KD: ketogenic diet
LCT: long‐chain triglycerides
MAD: modified atkins diet
MCT: medium‐chain triglycerides
SLC2A1: solute carrier family 2 member 1

## 1 INTRODUCTION

Glucose transporter type 1 deficiency syndrome (GLUT1DS) is characterised by early onset epilepsy, movement disorders, and cognitive impairment.^8^ Movement disorders may present as constant and/or paroxysmal disorders, including paroxysmal episodic dyskinesia and paroxysmal eye movement disorders.^8^, ^11^ GLUT1DS is caused by mutations in *SLC2A1*, which result in deficient glucose transport to the brain.^8^ Since the disease elucidation in 1991, increasing numbers of GLUT1DS patients have been treated with a ketogenic diet (KD).^9^ In the absence of glucose, ketones serve as an alternative energy source for the brain. In classical KD, 90% of calories consist of long‐chain triglycerides (LCT). Alternative dietary treatments involve lower fat‐to‐carbohydrate ratios, medium‐chain triglycerides (MCT) supplementation, and modified Atkins diet (MAD), with only carbohydrate restriction.^7^, ^9^ Positive effects of dietary treatment on GLUT1DS symptoms have been described in case reports and series, but larger studies are lacking, putatively due to the rareness of GLUT1DS and absence of alternative therapies.^7^, ^8^ Combined with adherence difficulties, this might explain why not all GLUT1DS patients receive dietary treatment (worldwide registry: 77/181 [43%] of patients).^6^ The current 25‐year experience with dietary treatment for GLUT1DS now allows extensive evaluation of treatment effects.

## 2 METHODS

Literature was searched up to January 2019 in PubMed, Embase and through cross‐referencing (Supporting Information 1). Studies describing clinical effects of dietary treatment for GLUT1DS were included and data from individual GLUT1DS patients with dietary treatment were aggregated as previously reported for rare diseases.^5^ To avoid duplicate reporting, the most representative of studies suspected to report overlapping patients was selected and data of identical patients described in different studies were combined. If data could not be related to individual cases, percentages were used for registration of (side) effects.

GLUT1DS was defined as presence of epilepsy, movement disorder, and/or cognitive impairment and cerebrospinal fluid (CSF)‐to‐blood glucose ratio <0.50 and/or a *SLC2A1* mutation.

Effects were categorised as ‘deterioration’, including development of new symptoms; ‘no change’, only including patients with pre‐existing symptoms; ‘improvement’; or ‘disappearance of symptoms’. For statistical analyses, treatment response was categorised as ‘improvement’, combining improvement and disappearance of symptoms, and ‘disappearance’ of symptoms. ‘Cognitive improvement’ was defined as increased IQ ≥5 points and improved learning and school performances.

### 2.1 Statistical analyses

To determine host and nutritional factors associated with treatment response, univariate and multivariable logistic regression analyses were performed. The multivariable model included the possible confounding variables age at treatment initiation, CSF‐to‐blood glucose ratios, sex and, for epilepsy, diet composition. For movement disorders, diet composition could not be included in multivariable analysis due to uneven distribution of diets between patient groups. Ketosis and time until observation of effect could not be included in multivariable analyses due to insufficient data. Similarly, information and patient numbers were insufficient to analyse factors influencing treatment response for cognition and factors influencing deterioration of symptoms. *P*‐values <.05 were considered statistically significant.

## 3 RESULTS

**Figure 1:**
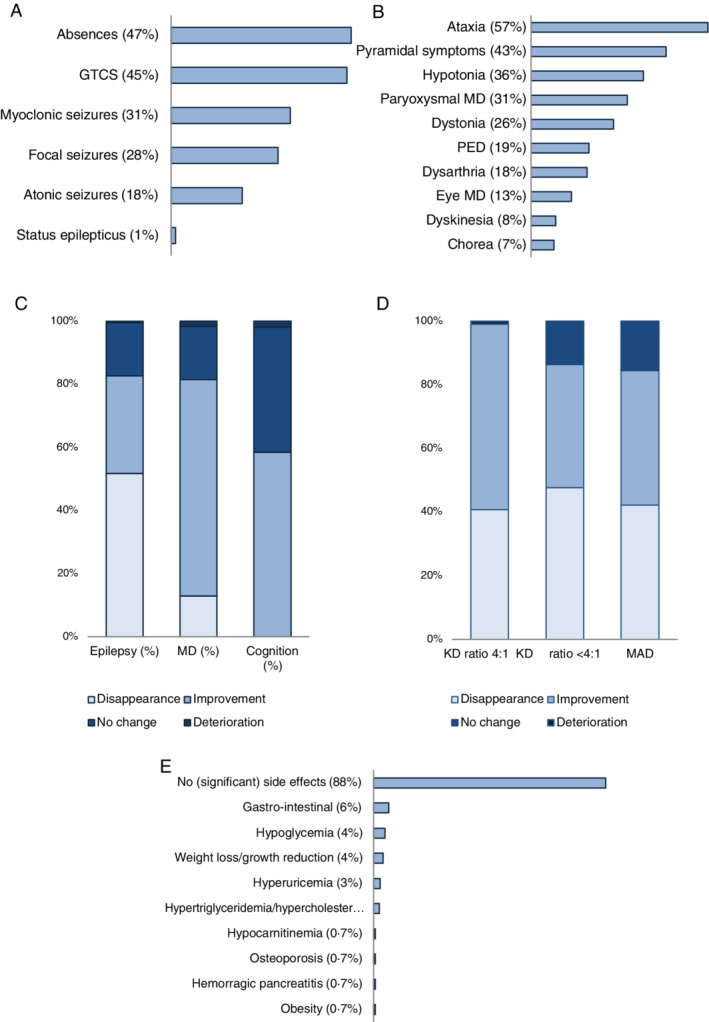
Symptoms, dietary effect, influence of diet composition, and side effects in reported GLUT1DS patients. Reported type of epileptic seizures (n = 185), A and movement disorders (n = 178), B. Clinical effect of dietary treatment on epilepsy (n = 238), movement disorders (n = 132), and cognition (n = 58), C. The effect of dietary composition (classical KD [n = 82], KD with lower ratios [n = 47], and MAD [n = 27] on epilepsy in percentage of patients), E. Reported side effects associated with dietary treatment, F. GLUT1DS, glucose transporter type 1 deficiency syndrome; GTCS, generalised tonic/clonic seizures; KD, ketogenic diet; MAD: modified Atkins diet; MD, movement disorder; PED, paroxysmal episodic dyskinesia

We identified 270 GLUT1DS patients using dietary treatment in 60 case reports, series, and surveys (Supporting Information 2). Mean treatment and follow‐up duration were 49 and 53 months, respectively (Table 1). Prior to treatment, epilepsy was reported in 82%, movement disorders in 66%, and cognitive impairment in 59% of patients (Figure 1A,B).

**Table 1:** Patient characteristics

Characteristics		Number of patients (total = 270)
Male:Female ratio (n:n)	1.1:1 (137:129)	266
Age at presentation in months, mean (SD)	17.1 (25.4)	126
Microcephaly in % (*n*)	Yes: 20.7 (*56*)	270
	No: 41.5 (*112*)	
	NR: 37.8 (*102*)	
CSF‐to‐blood glucose ratio, mean (SD)	0.37 (0.073)	174
CSF glucose (mmol/l), mean (SD)	2.0 (0.78)	136
Mutation in *SLC2A1* gene in % (n)	Yes: 89.6 (*237*)	270
	No: 1.9 (*5*)	
	NR: 10.4 (*28*)	
Age at initiation diet in months, mean (SD)	71.3 (52.0)	262
Duration of diet in months, mean (SD)	48.5 (34.8)	156
Duration of follow‐up in months, mean (SD)	52.7 (31.5)	126
Type of diet in % (*n*)	cKD: 30.7 (*83*)	270
	KD ratio < 4:1: 18.9 (*51*)	
	MAD: 11.9 (*32*)	
	KD ratio unreported: 38.5 (*104*)	
Reached ketosis in % (*n*)	Yes: 19.6 (*53*)	270
	No: 6.7 (*18*)	
	NR: 73.7 (*199*)	

	Epilepsy	Movement disorders	Development delay and/or cognitive impairment
Presence of symptoms in % (*n*)	Yes: 82.2 (*222*)	Yes: 65.9 (*178*)	Yes: 59.3 (*160*)
	No: 8.5 (*23*)	No: 6.7 (*18*)	No: 4.4 (*12*)
	NR: 9.3 (*25*)	NR: 27.4 (*74*)	NR: 36.3 (*98*)
Time until observation of effect on symptoms in % (*n*)	Within days: 56.9 (*33*)	Within days: 0.0 (*0*)	Within days: 0.0 (*0*)
	Within weeks: 24.1 (*14*)	Within weeks: 35.0 (*7*)	Within weeks: 12.0 (*3*)
	Within months: 15.5 (*9*)	Within months: 55.0 (*11*)	Within months: 64.0 (*16*)
	Within years: 3.4 (*2*)	Within years: 1.0 (*2*)	Within years: 24.0 (*6*)
	Total: n = 58	Total: n = 20	Total: n = 25

*Note*: Cognitive impairment was categorised according to the IQ classification score in ‘normal’ cognitive function (IQ > 80), ‘mild’ cognitive impairment (IQ 50‐80), ‘moderate’ cognitive impairment (IQ 35‐50), and ‘severe’ cognitive impairment (IQ <35). We defined slightly retarded as ‘mild’ cognitive impairment. When only learning difficulties were reported, cognitive functioning was categorised as ‘normal’.

Abbreviations: CSF, cerebrospinal fluid; cKD, classical ketogenic diet; KD, ketogenic diet; NR, not reported; MAD, modified Atkins diet.

Effect of dietary treatment on epilepsy was described for 230 patients (Figure 1C). For the majority (83%), epileptic seizures disappeared (52%) or decreased (31%). Epilepsy remained unchanged in 17% and deteriorated in only 0.4%. Multivariable analysis including age at treatment initiation, CSF‐to‐blood glucose ratio, sex and diet composition as independent variables only showed a significant effect for the age at treatment initiation, which was more effective in resolving epilepsy after early treatment initiation when compared to later treatment initiation (n = 147; *P* = .04, Table 2A).

**Table 2:** Univariate and multivariable analysis of factors associated with treatment effect

(A)		Improvement of epilepsy		Disappearance of epilepsy	
		Univariate analysis	Multivariable analysis (n = 147)	Univariate analysis	Multivariable analysis (n = 147)
Characteristics	n*	OR (95% CI)	OR (95% CI)	OR (95% CI)	OR (95% CI)
Age at initiation	165	1.01 (1.00‐1.02)*	1.01 (0.99‐1.01)	1.01 (1.00‐1.01)*	1.01 (1.00–1.01)*
Diet composition					
Classical KD	18	1	1	1	1
MAD	24	3.40 (0.35‐33.4)	2.15 (0.19‐24.9)	0.89 (0.26‐3.10)	0.46 (0.11‐1.98)
Lower ratios KD	43	2.24 (0.24‐20.6)	0.98 (0.86‐11.1)	0.70 (0.23‐2.13)	0.60 (0.17‐2.10)
Unkown	81	2.75 (0.33‐22.7)	1.82 (0.21‐16.1)	0.72 (0.25‐2.04)	0.60 (0.19‐1.88)
CSF/blood glucose ratio^a^	148	14.1 (0.77‐258)	12.7 (0.61‐265)	4.10 (0.87‐19.3)	3.37 (0.68‐16.75)
Sex					
Female	83	1	1	1	1
Male	84	0.87 (0.35‐2.18)	1.09 (0.38‐3.15)	1.66 (0.90‐3.07)	1.68 (0.84‐3.35)

(B)		Improvement of movement disorders		Disappearance of movement disorders	
		Univariate analysis	Multivariable analysis (n = 104)	Univariate analysis	Multivariable analysis (n = 104)
Characteristic	n*	OR (95% CI)	OR (95% CI)	OR (95% CI)	OR (95% CI)
Age at initiation	120	0.99 (0.99‐1.00)	0.99 (0.99–1.00)	0.99 (0.98‐1.00)	0.99 (0.98‐1.00)
CSF/blood glucose ratio^a^	104	0.03 (0.00‐0.32)*	0.03 (0.00‐0.34)*	0.03 (0.00‐0.73)	0.05 (0.00‐1.37)
Sex					
Female	54	1	1	1	1
Male	69	0.66 (0.27‐1.65)*	0.66 (0.24‐1.87)	2.39 (0.81‐7.05)	4.55 (1.2‐17.0)*

Abbreviations: KD, ketogenic diet; MAD, modified Atkins diet; n, number of patients, n*, number of patients included in univariate analysis.

* *P* < .05.

a Log ratio was used for statistical analysis of CSF‐to‐blood glucose ratio.

Movement disorders disappeared (13%) or improved (69%) in 82%, remained unchanged in 17% and deteriorated in 1.6% of 127 patients (Figure 1C). Multivariable analysis including age at treatment initiation, CSF‐to‐blood glucose ratio and sex as independent variables showed significantly more improvement of movement disorders in patients with higher than lower CSF‐to‐blood glucose ratios (n = 104; *P* = .01) and more resolution of movement disorders in girls (n = 104 [NB only 14 patients in this subgroup]; *P* = .004, Table 2B).

Cognition improved in 59% of 58 patients, remained stable in 40%, and deteriorated in 1.7% (Figure 1C). IQ‐scores improved in nine of 10 patients with measurements before and during treatment from a mean IQ of 50.6 to 55.4 after mean treatment duration of 7.1 months.

Microcephaly was reported in 34% of 168 patients (Table 1) and the effect of dietary treatment on microcephaly only for 11 patients. For two of these patients, head growth improved during dietary treatment, but all patients remained microcephalic after a mean follow‐up of 39 months.

**Figure 2:**
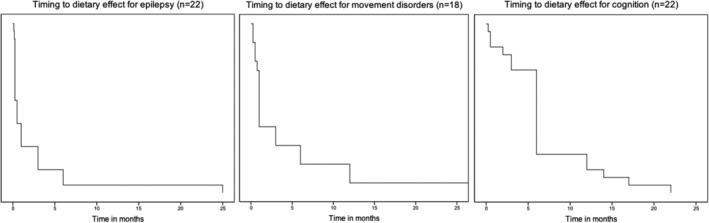
Timing to dietary effect for epilepsy (n = 22), movement disorders (n = 18), and cognition (n = 22) in a Kaplan‐Meier curve

Treatment effect was seen within days (57% of 58 patients) or weeks (24%) for epilepsy, after weeks (35% of 20 patients) or months (55%) for movement disorders, and after months (64% of 25 patients) or years (24%) for cognition (Table 1 and Figure 2).

Side effects were explicitly evaluated in 173 of 270 patients; 81% reported absence of significant side effects, 6% gastrointestinal symptoms, 4% hypoglycaemia, and 4% weight loss or failure to thrive (Figure 1E). About 18% of 270 patients reported compliance problems.

Ten patients discontinued treatment due to absence of beneficial effects (n = 2), or compliance problems and side effects (n = 8). The effect of treatment discontinuation on epilepsy was reported for only five patients, and this led to deterioration in four of them. The effect of treatment discontinuation on movement disorders was reported in three patients and led to deterioration in two of them. For nine patients, symptoms strongly depended on compliance and degree of ketosis.

## 4 DISCUSSION

Reviewing 25 years of experience with dietary treatment in 270 GLUT1DS patients, we discerned a strong positive effect on all GLUT1DS symptoms. Epilepsy improved in 83% and remained unchanged in 17%, movement disorders improved in 82% and remained unchanged in 17%, and cognition improved in 59% and remained unchanged in 40% of patients.

Most patients noticed treatment effects within days or weeks for epilepsy and after weeks or months for movement disorders and cognition. This information is important for expectation management and compliance. Early treatment initiation was favourable for treatment of epilepsy. Disease severity, as reflected by CSF‐to‐blood glucose ratio, affected treatment outcome for movement disorders, with most beneficial effects in less severe patients. This concurs with our experience that movement disorders are difficult to treat, but milder forms are more amenable to treatment. These findings might imply that dietary treatment is most effective in restoring the acute cerebral energy deficit causing epilepsy. The more protracted effect on movement disorders and cognition may reflect another pathophysiological mechanism.

Adherence to the strict KD remains difficult. Compliance problems were reported in 18%, and this might well represent an underestimation due to reporting and publication bias. The strong benefits of dietary treatment and the finding of higher efficacy upon treatment adherence in a previous review of GLUT1DS patients underscore the importance of compliance.^6^ Although diets with lower fat‐to‐carbohydrate ratios were effective in treating epilepsy in our study, higher fat‐to‐carbohydrate ratios were previously positively correlated with efficacy.^6^

Side effects were minimal, also when compared with KD treatment of epilepsy.^3^ This may relate to more heterogeneity and other organ involvement in epilepsy patients. Alternatively, side effects may have been underestimated because they were reported for only 64% of patients. Reporting bias, duplicate patients and the relatively short follow‐up may have influenced our findings in general. Moreover, relating our results to the natural course of GLUT1DS is difficult, because dietary treatment has constituted the cornerstone of treatment since the first description.^4^ A long‐term follow‐up study of GLUT1DS patients (13%: dietary treatment; 47%: unknown) reported that movement disorders increased with age, cognition remained stable and epilepsy decreased during adolescence.^10^ Our results show that with treatment started at a mean age of 6 years, epilepsy decreased long before adolescence and movement disorders and cognition improved in most patients. Benefits of dietary treatment are further underscored by deterioration of symptoms upon poor compliance, low ketosis, or treatment discontinuation. Moreover, positive effects of early treatment initiation in sibs illustrate the potential of dietary treatment in preventing progressive deterioration.^2^, ^12^ Despite these positive effects of dietary treatment for most patients, recently a retrospective study analysed seven patients with KD treatment failure.^1^ Compared with our patients and previously reported cohorts, this cohort of patients was characterised by a later onset of seizures (mean age: 43 months compared to 11.5 months in our cohort), and advanced age at diagnosis and initiation of dietary treatment. Treatment failure in these older patients at diagnosis correlates with the finding that younger age at diagnosis increased the probability of KD efficacy in the worldwide GLUT1DS registry (https://www.g1dregistry.org),^6^ and might imply that treatment should be initiated promptly to prevent irreversible damage.

In conclusion, analysis of the reported 25‐year experience with dietary treatment for GLUT1DS reveals strong favourable effects on all symptoms and minimal side effects, underscoring the urgency to treat GLUT1DS with KD and adhere to the strict diet. We encourage early diagnosis or screening to start treatment promptly.

## CONFLICT OF INTEREST

M. Schwantje, Dr. Verhagen, Dr. van Hasselt, and Dr. Fuchs declare to have no potential conflicts of interests. None of the authors have accepted reimbursements, fees, funds, or salaries from an organisation that may in any way gain or lose financially from the results reported in this manuscript. None of the authors have any competing interests regarding relevant financial activities outside the submitted work, intellectual property or any other relationships.

## AUTHOR CONTRIBUTIONS

M.S. and S.A.F. were involved in conception and design of the study, and acquisition of data; L.M.V., M.S., P.M.H., and S.A.F. were involved in analysis and interpretation of data. M.S. and S.A.F. drafted the manuscript. L.M.V., M.S., S.A.F., and P.M.H. were involved in reviewing and editing the manuscript. All authors have given final approval of the version to be published. M.S. and S.A.F. take responsibility for the collection of data, the analyses, interpretation and publication.

## ETHICS STATEMENT

This article only describes the analysis of already reported patient characteristics (after ethical approval by the authors centers—mostly explicitly reported). This article does not contain any studies with human or animal subjects performed by the any of the authors.

## INFORMED CONSENT

Only patients whose dietary treatment effects had already been reported in literature were included in our study; by including 270 anonymous patients and presenting clinical effects group wise, none of the individual patients can be traced and additional informed patient consents were not obtained.

## Supporting information

Supplement 1: Flow chart of the study selection process.
Supplement 2: Reference list of included articles.

## Data Availability

Data used for the analyses performed for this article will be shared upon (reasonable) request to the corresponding author.
