# Supplementary material for: Glucose transporter type 1 deficiency syndrome and the ketogenic diet
Source: J Inherit Metab Dis. 2019 Nov 13;43(2):216–22. doi: 10.1002/jimd.12175 (PMC7078900; doi:10.1002/jimd.12175)
Supplement: Supplementary file 1 — Supplement 1: Flow chart of the study selection process. Supplement 2: Reference list of included articles. [file JIMD-43-216-s001.docx]

**Supplemental data**

**Supplement 1: Flow chart of the study selection process**

**
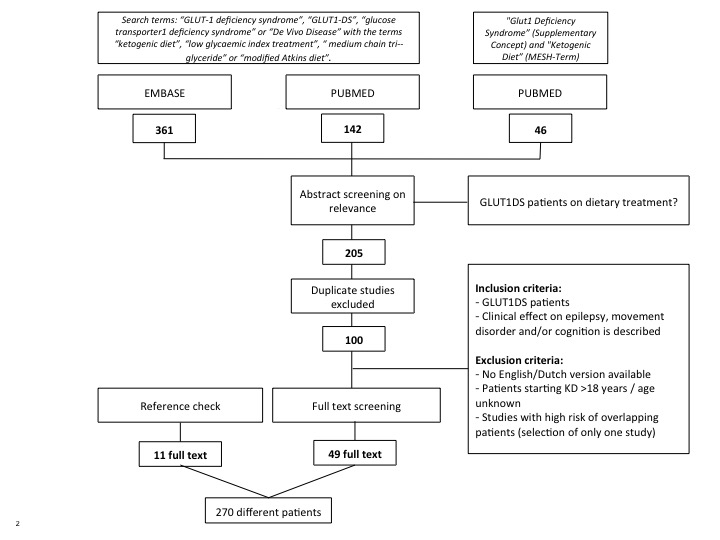
**

**Supplement 2: Reference list of included articles**

1. Ismayilova N, Hacohen Y, MacKinnon AD, Elsmslie F, Clarke A (2018) GLUT-1 deficiency presenting with seizures and reversible leukencephalopathy on MRI imaging. Eur J Paediatr Neurol 22(6):1161-1164
2. Van Kan EM, Panis B (2018) Exercise and fasting induced movement disorder in children: thing of the GLUT1 deficiency syndrome. Ned Tijdschr Geneeskd 162.
3. Reis S, Matias J, Machado R, Monteiro JP (2018) Paroxysmal ocular movements – an early sign of Glut1 deficiency syndrome. Metab Brain Dis 33(4):1381-1383.
4. Tchapyjnikov D, Mikati MA (2018) Azetolamide-responsive Episodic Ataxia Without Baseline Deficits or Seizures Secondary to GLUT1 Deficiency: A Case Report and Review of the Literature. Neurologist 23(1):17-18.
5. Panandikar GA, Ravat SH, Ansari RR, Desai KM (2018) Rare and treatable cause of early-onset refractory absence seizures. Metabolic Brain Disease 33(4):1381-1383.
6. Braakman HMH, Engelen M, Nicolai J, Willemsen MA (2018) Stroke mimics add to the clinical spectrum of GLUT1 deficiency syndrome. J Neurol Neurosurg Psychiatry 89(6):668-670.§
7. Shibata T, Kobayashi K, Yoshinaga H, Ono H, Shinpo M, Kagitani-Shimono K(2017) Another Case of Glucose Transporter 1 Deficiency Syndrome with Periventricular Calcification, Cataracts, Hemolysis, and Pseudohyperkaliemia. Neuropaediatrics 48:390­–393.
8. Ramm-Pettersen A, Nakken KO, Haavardsholm KC, Selmer KK (2017) GLUT1-deficiency syndrome: Report of a four-generation Norwegian family with a mild phenotype. Epilepsy Behav 70:1–4.
9. Juozapaite S, Praninskiene R, Burnyte B, Ambrozaityte L, Skerliene B (2017) Novel mutation in a patient with late onset GLUT1 deficiency syndrome. Brain Dev 39:352–355.
10. Pearson TS, Pons R, Engelstad K, Kane SA, Goldberg ME, De Vivo DC (2017) Paroxysmal eye-head movements in Glut1 deficiency. Neurology 88(17):1666-1673
11. Pellegrin S, Cantalupo G, Opri R, Dalla Bernardina B, Darra F (2017) EEG findings during “paroxysmal hemiplegia” in a patiënt with GLUT1-deficiency. Eur J Paediatr Neurol 21(3)580-582.
12. Amalou S, Gras D, Ilea A, et al (2016) Use of modified Atkins diet in glucose transporter type 1 deficiency syndrome. Dev Med Child Neurol 58:1193–1199.
13. Fuji T, Ito Y, Takashahi S et al (2016) Outcome of ketogenic diets in GLUT1 deficiency syndrome in Japan: A nationwide survey. Brain Dev 38:628–637.
14. Appavu B, Mangum T, Obeid M (2015) Glucose Transporter 1 Deficiency: A treatable Cause of Opsoclonus and Epileptic myoclonus. Pediatr Neurology 53:364–366.
15. Gumus H, Bayram AK, Kardas F, et al (2015) The effects of ketogenic diet on seizures, cognitive functions, and other neurological disorders in Classical phenotype of glucose transporter 1 deficiency syndrome. Neuropediatrics 46:313–320.
16. Sen S, Keough K, Gibson J (2015) Clinical Reasoning: Novel GLUT1-DS mutation. Neurology 84:e111–114.
17. Szczepanik E, Terczyńska I, Kruk M, et al (2015) Glucose transporter type 1 deficiency due to SLC2A1 gene mutations - a rare but treatable cause of metabolic epilepsy and extrapyramidal movement disorder; own experience and literature review. Dev Period Med 19:454–463.
18. Chenouard A, Vuillaumier-Barrot S, Seta N. Kuster A (2015) A Cause of Permanent Ketosis: GLUT-1 Deficiency. JIMD Resp 18:79–83.
19. Ramm-Pettersen A, Stabell KE, Nakken KO, Selmer KK (2014) Does ketogenic diet improve cognitive function in patients with GLUT1-DS? A 6- to 17-month follow-up study. Epilepsy Behav 39:111–115.
20. Haberlandt E, Karall D, Jud V, et al (2014) Glucose transporter type 1 deficiency syndrome effectively treated with modified atkins diet. Neuropediatrics 45:117–119.
21. Vykuntaraju KN, Bhat S, Sanjay KS, Govindaraju M (2014) Symptomatic West Syndrome Secondary to Glucose Transporter-1 (GLUT1) Deficiency with Complete Response to 4:1 Ketogenic Diet. Indian J Pediatr 81:934–936.
22. Leen WG, Taher M, Verbeek MM, Kamsteeg EJ, van de Warrenburg BP, Willemsen MA (2014) GLUT1 deficiency syndrome into adulthood: A follow-up study. J Neurol 261:589–599.
23. Posar A, Santucci M (2014) Unusual phenotype of glucose transport protein type 1 deficiency syndrome: A case report and literature review. J Pediatr Neurosci 9:36–38.
24. Mohammad SS, Coman D, Calvert S (2014) Glucose transporter 1 deficiency syndrome and hemiplegic migraines as a dominant presenting clinical feature. J Paediatr. Child Health 50:1025–1026.
25. Wolking S, Becker F, Bast T et al (2014) Focal epilepsy in Glucose transporter type 1 (Glut1) defects: Case reports and a review of literature. J. Neurol 261:1881–1886.
26. Shiohama T, Fujii K, Takahashi S, Nakamura F, Kohno Y (2013) Reversible white matter lesions during ketogenic diet therapy in glucose transporter 1 deficiency syndrome. Pediatr Neurol 49:493–496.
27. Leen WG, Mewasingh L, Verbeek MM, Kamsteeg EJ, van de Warrenburg BP, Willemsen MA (2013) Movement disorders in GLUT1 deficiency syndrome respond to the modified Atkins diet. Mov. Disord 28:1439–1442.
28. Ramm-Pettersen A, Nakken KO, Skogseid IM, et al (2013) Good outcome in patients with early dietary treatment of GLUT-1 deficiency syndrome: results from a retrospective Norwegian study. Dev Med Child Neurol 55:440–447.
29. Woo SB, Lee KH, Kang HC, Yang H, De Vivo DC, Kim SK (2012) First report of glucose transporter 1 deficiency syndrome in Korea with a novel splice site mutation. Gene 506:380–382.
30. Gramer G, Wolf NI, Vater D, et al (2012) Glucose Transporter-1 (GLUT1) Deficiency Syndrome: Diagnosis and Treatment in Late Childhood. Neuropediatrics 43:168–171.
31. Bawazir WM, Gevers EF, Flatt JF, et al (2012) An infant with pseudohyperkalemia, hemolysis, and seizures: cation-leaky GLUT1-deficiency syndrome due to a SLC2A1 mutation. J Clinical Endocrinol Metabol 97:e987–993.
32. Pong AW, Geary BR, Engelstad KM, Natarajan A, Yang H, De Vivo DC (2012) Glucose transporter type I deficiency syndrome: epilepsy phenotypes and outcomes. Epilepsia 53:1503–1510.
33. Koy A, Assmann B, Klepper J, Mayatepek E (2011) Glucose transporter type 1 deficiency syndrome with carbohydrate-responsive symptoms but without epilepsy. Dev Med Child Neurol 53:1154–1156.
34. Gaspard N, Suls A, Vilain C, De Jonghe P, Van Bogaert P (2011) Benign myoclonic epilepsy of infancy as the initial presentation of glucose transporter-1 deficiency. Epileptic disorders 13:300–303.
35. Anand G, Padeniya A, Hanrahan D, et al (2011) Milder phenotypes of glucose transporter type 1 deficiency syndrome. Dev Med Child Neurol 53:664–668.
36. Fung EL, Ho YY, Hui J, et al (2011) First report of GLUT1 deficiency syndrome in Chinese patients with novel and hot spot mutations in SLC2A1 gene. Brain Dev 33:170–173.
37. Byrne S, Kearns J, Carolan R, et al (2011) Refractory absence epilepsy associated with GLUT-1 deficiency syndrome. Epilepsia 52:1021–1024.
38. Urbizu A, Cuenca-Léon E, Raspall-Chaure M, et al (2010) Paroxysmal exercise-induced dyskinesia, writer's cramp, migraine with aura and absence epilepsy in twin brothers with a novel SLC2A1 missense mutation. J Neurol Sci 295:110–113.
39. Veggiotti P, Teutonico F, Alfei E, et al (2010) Glucose transporter type 1 deficiency: Ketogenic diet in three patients with atypical phenotype. Brain Dev 32:404–408.
40. Leen WG, Klepper J, Verbeek M.M, et al (2010) Glucose transporter-1 deficiency syndrome: the expanding clinical and genetic spectrum of a treatable disorder. Brain 133:655–670.
41. Klepper J, Scheffer H, Elsaid MF, Kamsteeg EJ, Leferink M, Ben-Omran T (2009) Autosomal recessive inheritance of GLUT1 deficiency syndrome. Neuropediatrics 40:207–210.
42. Pérez-dueñas B, Prior C, Ma Q, et al (2009) Childhood chorea with cerebral hypotrophy: A treatable GLUT1 energy failure syndrome. Arch Neurol 66:1410–1414.
43. Slaughter L, Vartzellis G, Arthur T (2009) New GLUT-1 mutation in a child with treatment-resistant epilepsy. Epilepsy Res 84:254–256.
44. Rotstein M, Doran J, Yang H, Ullner PM, Engelstad K, De Vivo DC (2009) Glut 1 deficiency and alternating hemiplegia of childhood. Neurology 73:2042–2044.
45. Harris ML, Patel H, Garg BP (2008) Intractable Seizures, Developmental Delay, and the Ketogenic Diet. Semin Pediatr Neurol 15:209–211.
46. Joshi C, Greenberg CR, De Vivo D, Dong Wang, Chan-Lui W, Booth FA (2008) GLUT1 deficiency without epilepsy: Yet another case. J Child Neurol 23:832–834.
47. Suls A, Dedeken P, Goffin K, et al (2008) Paroxysmal exercise-induced dyskinesia and epilepsy is due to mutations in SLC2A1, encoding the glucose transporter GLUT1. Brain 131:1831–1844.
48. Roulet-Perez E, Ballhausen D, Bonafé L, Cronel-Ohayon S, Maeder-Ingvar M (2008) Glut-1 deficiency syndrome masquerading as idiopathic generalized epilepsy. Epilepsia 49:1955–1958.
49. Klepper J, Engelbrecht V, Scheffer H, Van der Knaap MS, Fiedler A (2007) GLUT1 Deficiency With Delayed Myelination Responding to Ketogenic Diet. Pediatr Neurol 37:130–133.
50. Friedman JR, Thiele EA, Wang D (2006) Atypical GLUT1 deficiency with prominent movement disorder responsive to ketogenic diet. Mov. Disord 21:241–245.
51. Coman DJ, Sinclair KG, Burke CG, et al (2006) Seizures, ataxia, developmental delay and the general paediatrician: glucose transporter 1 deficiency syndrome. J Paediatr Child Health 42:263–267.
52. Klepper J, Scheffer H, Leiendecker B, et al (2005) Seizure control and acceptance of the ketogenic diet in GLUT1 deficiency syndrome: A 2- to 5-year follow-up of 15 children enrolled prospectively. Neuropediatrics 36:302–308.
53. Wang D, Pascual JM, Yang H, et al (2005) Glut-1 deficiency syndrome: Clinical, genetic, and therapeutic aspects. Ann Neurol 57:111–118.
54. Klepper J, Diefenbach S, Kohlschütter A, Voit T (2004) Effects of the ketogenic diet in the glucose transporter 1 deficiency syndrome. Prostaglandins Leukot Essent Fatty Acids 70:321–327.
55. Overweg-Plandsoen WG, Groener JE, Wang D, et al (2003) GLUT-1 deficiency without epilepsy - An exceptional case. J Inherit Metab Dis 26:559–563.
56. Gordon N, Newton RW (2003) Glucose transporter type1 (GLUT-1) deficiency. Brain Dev 25:477–480.
57. De Vivo DC, Leary L, Wang D (2002) Glucose transporter 1 deficiency syndrome and other glycolytic defects. J Child Neurol 17 Suppl 3:3S15–23.
58. Brockmann K, Wang D, Korenke CG, et al (2001) Autosomal dominant Glut-1 deficiency syndrome and familial epilepsy. Ann Neurol 50:476–485.
59. Boles RG, Seashore MR, Mitchell WG, Kollross PR, Mofidi S, Novotny EJ (1999) Glucose transporter type 1 deficiency: a study of two cases with video-EEG. Eur J Paediatr 158:978–983.
60. De Vivo DC, Trifiletti RR, Jacobsen RI, Ronen GM, Behmand RA, Harik SI (1991) Defective glucose transport across the blood-brain barrier as a cause of persistent hypoglycorrhachia, seizures, and developmental delay. N Engl J Med 325:703–709.
